# Assessment of the Speed Management Impact on Road Traffic Safety on the Sections of Motorways and Expressways Using Simulation Methods

**DOI:** 10.3390/s20185057

**Published:** 2020-09-05

**Authors:** Jacek Oskarbski, Tomasz Kamiński, Kyandoghere Kyamakya, Jean Chamberlain Chedjou, Karol Żarski, Małgorzata Pędzierska

**Affiliations:** 1 Faculty of Civil and Environmental Engineering, Gdansk University of Technology, 80-233 Gdańsk, Poland; karol.zarski@pg.edu.pl; 2 Motor Transport Institute, 03-301 Warszawa, Poland; tomasz.kaminski@its.waw.pl (T.K.); malgorzata.pedzierska@its.waw.pl (M.P.); 3 Institute for Smart Systems Technologies, University Klagenfurt, A9020 Klagenfurt, Austria; kyandoghere.kyamakya@aau.at (K.K.); jean.chedjou@aau.at (J.C.C.)

**Keywords:** variable speed limits, intelligent transportation systems, ITS services, driving simulator studies, traffic modelling, surrogate safety measures

## Abstract

Methods used to evaluate the impact of Intelligent Transport System (ITS) services on road safety are usually based on expert assessments or statistical studies. However, commonly used methods are challenging to apply in the planning process of ITS services. This paper presents the methodology of research using surrogate safety measures calculated and calibrated with the use of simulation techniques and a driving simulator. This approach supports the choice of the type of ITS services that are beneficial for traffic efficiency and road safety. This paper presents results of research on the influence of selected scenarios of variable speed limits on the efficiency and safety of traffic on the sections of motorways and expressways in various traffic conditions. The driving simulator was used to estimate the efficiency of lane-keeping by the driver. The simulation traffic models were calibrated using driving simulator data and roadside sensor data. The traffic models made it possible to determine surrogate safety measures (number of conflicts and their severity) in selected scenarios of using ITS services. The presented studies confirmed the positive impact of Variable Speed Limits (VSLs) on the level of road safety and traffic efficiency. This paper also presents recommendations and plans for further research in this area.

## 1. Introduction

Variable Speed Limit (VSL) systems have been implemented in many countries as a method of improving traffic flow and road safety. Upon completion of the data analysis process, the recommended speed limits are dynamically updated, with new messages being displayed on Variable Message Signs (VMSs) to influence driver behaviour. The VSL algorithms are usually based on speed, occupancy and volume variables. The desired speed is reduced upstream to limit the spread of shock waves at critical values of the variables defined in the algorithm. Properly designed VSL systems reduce the number and severity of accidents, travel time and emissions by harmonising traffic flow speed [1,2,3]. The benefits of VSL were also presented by Papageorgiou et al. [4] and by Abdel-Aty et al. [5], where the development of a crash model was described. Li et al. [6] presented the impact of VSL on reducing the number of secondary collisions in poor visibility conditions. For this purpose, Li used a modified car-following model. VSL systems contribute to improving traffic safety by reducing the speed difference between vehicles and minimising the speed variation, resulting in less frequent lane changes and sudden braking.

In 2017–2019, 2078 accidents occurred on Polish motorways and expressways. As a result, 2965 people were injured (including 773 seriously injured) and 295 people died [7]. Further expansion of the high-speed road network in Poland, and thus shifting the majority of the traffic to expressways, may consequently lead to an increase in the number of collisions and accidents, as well as the number of fatalities and injuries on these roads.

In 2011–2015, 2339 accidents (1.3% of the total number of accidents) occurred on motorways and expressways within a length of 3194.55 km (17% of the length of national roads, 1.1% of all roads in Poland). They caused 402 deaths (2.3% of the total number of fatalities) and 3443 injuries (1.6% of the total). The analysis of trends in 2011–2015 indicated the growing risk of being involved in an accident on motorways and expressways. Although these changes are caused by the development of the road network, the increase in the number of accidents and victims is more significant than the increase in the length of these roads. As the data show, motorways exceed expressways in terms of increased collision risk. The situation is even more worrying when compared to other national roads, where the risk has decreased significantly in the same period. The accident (number of accidents per 100 km) and victim (number of victims per 100 km) densities for motorways and expressways are much lower than on other national roads. Nevertheless, these rates on expressways and motorways are increasing, while on other roads a reduction is observed. Analysis of the data showed that there was a decrease in the severity of accidents (except for motorways). However, accidents on motorways (16 fatalities per 100 accidents) and expressways (15 fatalities per 100 accidents) were much more severe than on other roads (nine fatalities per 100 accidents). Among the accidents that occurred on motorways and expressways, rear-end collisions of vehicles were the most frequent (34.2 % of all accidents). Besides, drivers were found to exceed speed limits (19%), not maintain a safe distance (8%) and change lanes incorrectly (4%), leading to a collision. On motorways and expressways (similarly to other roads), road accidents most often occur during the day (64%) and up to 22% at night on unlit roads. Up to 64% of accidents occur in good weather conditions and, less frequently than on other national roads, accidents occur in adverse weather conditions: cloudy weather (18%), rainfall (11%), fog (2%) and snowfall (2%). It should be noted that on motorways and expressways, 72% of vehicles involved in accidents are passenger cars, while more often than on other national roads, Heavy Goods Vehicles (HGVs) are involved in accidents (23%) [8].

The authors of this paper identified the coverage of Polish motorways and expressways with different Intelligent Transport System (ITS) services based on data received from the National Road Administration (GDDKiA) under the project “The impact of the usage of Intelligent Transport System services on the level of road safety” (RID-4D) [9]. The studies of road traffic safety for motorway sections was carried out based on accidents and traffic volume data gathered in 2013–2015. For the assessment of road traffic safety, motorway sections without ITS services and sections with the implemented ITS services (primarily the provision of information to drivers via VMSs about weather conditions, adverse surface condition and related speed limits) were selected. Due to dispersed ITS services and the lack of a testing field and data for the case of using a series of variable message signs, it was not possible to apply statistical studies identifying the impact of an ITS service such as VSLs on the road safety level. The level of traffic safety was assessed using a risk estimation of individual involvement in an accident or becoming an accident victim. The risks were represented by the number of accidents and victims of these accidents per million vehicle kilometres travelled (VKT). The highest number of accidents per VKT was recorded on sections of motorways without ITS services. On sections equipped with ITS devices near urban areas, the risk of being a participant or a victim of an accident was higher than on motorway sections distant from highly urbanised areas. Similar relationships were observed for serious injuries and fatalities. The individual risk of being involved in an accident was 37% lower on motorways with ITS services than on motorways without such services. The number of fatalities per VKT was also 49% lower and the number of injuries was 26% lower. These results may suggest a positive influence of selected ITS services on the level of road traffic safety.

The results of the studies presented above [8,9] indicate that speed management measures (including Variable Speed Limits—VSLs) that can contribute to reducing collision risks are advisable to improve the level of road traffic safety. A significant proportion of accidents involving over-speed drivers and those who do not maintain a safe distance between vehicles require measures to harmonise traffic flow. The lack of sufficient data, and often, as in the case of Poland, the lack of test sites with fully functioning VSL systems, is the reason for using simulation methods to assess road traffic safety.

Statistical studies of accidents and their victims are a direct and widely used way to evaluate road safety issues. The condition for carrying out reliable statistical analyses is the use of data available over a longer period of several years and when the characteristics of the road and its surroundings do not change during this period. Insufficient statistical information creates a barrier in evaluating the safety level for the newly constructed roads or the roads in the designing or planning stages. The available data samples are small or evidence does not exist (including those for the accident rates). The above statement also applies to the planned road safety improvement measures to be implemented, including ITS services if they were not previously used and tested on the road [10].

The possibility to reliably assess the impact of planned ITS services on the functioning of the transport system is crucial considering the many services implemented on the roads which have successfully improved road safety and traffic efficiency. The accident data directly indicate both the structure and causes of the safety level. For each specific time and element of the transport system, the expected number of accidents or their victims can be estimated based on the known risk factors, consequences and exposure [11]. Many tools have been developed to select, process, analyse and visualise traffic accident data. Unfortunately, such tools do not take into account future changes that may affect the level of safety (including the development of new ITS services for vehicles or road infrastructure). The above statements demonstrate that the statistical analyses have little potential to assess ITS services, especially those recently introduced or planned for introduction. Besides, statistics are less useful for determining the causes of accidents, as they are rarely recorded in sufficient detail to conclude the complex chain of incidents preceding the accident [12]. Other safety assessment measures may be used when planning or introducing innovative road improvements.

One possibility is to use models to predict the number of accidents taking into account road characteristics (e.g., class of road) and traffic volume forecasts [13,14,15,16]. In 2010, the American Association of State Highway Transportation Officials (AASHTO) published the results of more than 10 years of research work carried out by many scientific centres and experts in the form of the Highway Safety Manual (HSM) [17]. The HSM presents a method of forecasting the number of accidents, victims and their costs. In 2012, the HSM method was extended [18]. The procedure of calculating the projected average number of accidents for individual elements of the motorway was presented for sections between interchanges, ramps, weaving and merging sections and also junctions within an interchange. Moreover, research was conducted using Bayesian methods [19,20,21,22,23] and advanced statistical techniques (e.g., classification and regression trees) to verify the results of analyses made using observations (traffic conflicts technique) [24,25,26]. Other ways of combining measures were also proposed to estimate the “level of service safety”, analogous to the traffic level of service (LOS) [27] or indicators for particular types of incidents (e.g., material loss incidents) [28]. Macroscopic measures of traffic flow were used in the proposed method.

Road safety researchers also use Surrogate Safety Measures (SSMs), derived from the theory of traffic conflicts. These measures are based on indirect indicators such as differences in speeds or estimated time to a collision of two interacting vehicles, to identify traffic conflicts and calculate their number. SSMs can be estimated based on the recording, analysis and comparison of trajectories and changes in movement dynamics of vehicles or other traffic users. Vehicle trajectories can be estimated by analysing data obtained from a real road section or junction (e.g., using video or RADAR techniques), data from a driving simulator or data obtained from simulations developed using traffic models.

This paper presents a novel methodology of research and assessment of road traffic safety using surrogate safety measures calculated based on simulation models and supported by driving simulator studies. In the presented studies, the Surrogate Safety Assessment Model (SSAM) was used for the first time in assessing the safety of ITS services, such as VSLs, with the use of surrogate safety measures. The added value is also the study and results of the location of variable message signs in the VSL system on a road section developed using the SSAM. The SSAM has so far been used to assess road traffic safety mainly due to the geometric parameters of the road and fixed traffic organisation measures [29], parameters of intersections (signalised or unsignalised) [30,31,32,33] or ramp metering ITS services [34]. The location and the number of variable message signs along road sections, depending on the traffic volume or accompanying road incidents, can influence the places where dangerous spots occur.

For this reason, it is important to identify such places in advance and limit their number to reduce risks. The surrogate safety measures are widely used in road safety analyses, however, to date the research has not been focused on such a broad approach as presented in this paper. This publication takes into account not only the impact of measures on individual elements of the road network (including types of intersections) but also on the entire network of co-existing roads (major road corridor). The method of calibrating the simulation model was also an added value. In the calibration process, data from sensors (inductive loops) of traffic measurement stations located on road cross sections were used. The data allowed us to develop cumulative distribution functions of key traffic flow variables in various traffic conditions and for different types of vehicles. The data from traffic measurement stations allowed us to develop functions in road cross sections (at selected points on each lane). To take into account the impact of the information displayed on the VMS, it was necessary to use data from the driving simulator, which made it possible to reproduce drivers’ behaviour (speed changes) along the road section upstream and downstream of the VMS. The dynamic model was used for traffic assignment in the road network.

The applied techniques can be used to assess road safety on planned roads and to determine changes in the safety level in case of planned road modernisation or the implementation of ITS services. Solutions in the field of ITS services on Polish motorways and expressways are currently being implemented on a large scale within the National Traffic Management System (KSZR). Poland lacks detailed guidelines for determining the structure of VSL systems (taking into account the location of VMSs and also sensors collecting data for traffic control) and the resulting distribution of VMSs, as well as the implementation of such a service on different roads. The presented research methodology, which takes into account different levels of traffic intensity and the occurrence of road incidents, may support the development of such guidelines.

## 2. Simulation Methods of Road Safety Assessment

Several methodologies have been proposed and applied in the scientific literature to collect and analyse data on SSMs and road users’ behaviour, including:

- naturalistic driving studies [35,36,37,38],
- site-based observation studies [39,40,41],
- microsimulation modelling studies [30,42,43],
- driving simulator studies [44,45,46,47].

The first two methodologies reflect the behaviour of road users in a real road environment, while the latter two can be considered as a controlled form of data collection in which researchers can manipulate and control traffic events [48]. This paper presents the application of methods based on simulations using microsimulation traffic models and a driving simulator.

### 2.1. Microsimulation Modelling Studies

The calculation of surrogate safety measures in microsimulation modelling studies is supported by traffic simulation models [29,49,50]. The simulation of traffic users’ behaviour when driving through a defined virtual road network requires the use of microsimulation models, which are computerised analytical tools [30]. The stochastic parameters adopted in the simulation models allow each traffic user to be treated as an individual unit and for the interaction between these individual units to be defined. [42]. These parameters make it possible to define individual preferences and trends in traffic users’ behaviour at a reasonable level of approximation. The Surrogate Safety Assessment Model (SSAM) is a tool for the collection and preliminary analysis of SSMs from microsimulation models. The SSAM is a post-processing tool using vehicle trajectories generated in microsimulation packages [51]. The use of SSMs and methods of assessing the road safety level on motorways and expressways require the consideration of three main types of events: rear-end collisions, side collisions (these two types of events require the participation of at least two vehicles) and single-vehicle accidents [52]. The use of commonly available simulation techniques is hampered by the need to take into account interactions with infrastructure elements in addition to the trajectory analysis in the case of incidents involving single vehicles. For the incidents involving one vehicle, the location of the accident (within the lane, off the road, within the sidewalk/emergency lane) and the severity of the accident were used to determine the accident topology [53]. The main benefit of research using microsimulation models is the possibility to evaluate the impact of the road infrastructure and ITS services on traffic safety proactively and without significant financial resources. The usefulness of most of the algorithms used in microsimulation models for safety assessments is limited due to focus on typical driver’s behaviour and the inability to take into account vehicle collision occurrences [43]. Some simplifications are inevitably necessary, even in the most advanced models. Therefore, the importance of results as a representation of actual road users’ behaviour could be discussed.

Simulation techniques at the microscopic level are used for the assessment of road safety based on surrogate measures. Golob et al. [53] indicated that the mean traffic volume, median speed and instantaneous deviations in the values of volume and speed significantly affect the possibility of an incident occurring. Xin et al. and Evans and Wasilewski showed that the most common cause of accidents is too small headways between vehicles [54,55]. Surrogate (indirect) safety measures might be speed and its variations, distances between vehicles in the traffic flow, traffic-related measures (including occupancy, traffic density, etc.) and lane change manoeuvres [30]. The most common measures used in simulation models are those used in traffic conflict theory [56]. The observation of sudden braking and avoidance manoeuvres makes it possible to identify traffic conflicts. The research conducted with the use of surrogate measures allows us to find a connection between the conflict and the real-life accident [57,58,59,60]. A traffic conflict is an observed situation in which two or more road users approach each other in space and time to such an extent that there is a risk of a collision if their movements (speed and direction) remain unchanged. In the face of the possibility of a collision, a fast, decisive manoeuvre of the vehicle, pedestrian or cyclist is required to avoid it. The underlying assumption of this method is that the greater the number of traffic conflicts, the more likely it is for an accident to occur. The method involves observing the elements of the road system and noting the conflict situations, i.e., those that could lead to the occurrence of an accident in particular areas of the road network (on a road section, in different parts of a junction).

Conflict is defined as an often-repeated behaviour of road users that can lead to an accident (e.g., braking too late, selection of an incorrect driving trajectory). Several basic measures, which are characteristic of traffic conflicts, have been proposed, e.g., Time To Collision (TTC), Deceleration Rate (DR) and the time interval between collision vehicles—leaving the collision point and arriving at the collision point (Post-Encroachment Time—PET). The most commonly used SSMs of traffic safety in the research was TTC followed by PET and their derivatives [30]. Surrogate measures may be used to determine the degree of significance of the conflict, which translates into the probability of accident severity [61]. The conflict technique allows us to obtain more data for analysis, but the parameters used to describe the manoeuvres/behaviour of drivers are indirect indicators of accident risk and the reduction of its severity.

Despite attempts to use the aggregated data to calculate the risk of incident occurrence, there are still uncertainties about the effectiveness of using the above-mentioned measures as risk measures [23]. However, it was estimated that the ratio between the frequency of conflicts calculated with the use of surrogate measures and the frequency of accidents is 20,000 to 1 [51]. SSMs can replace statistical measures for accidents and their victims. Observations of SSMs may be supplemented by behavioural observations and/or data from other fields, such as driving simulator tests. Traffic conflict theory is a proactive safety research method that can be used without waiting for accidents to happen as well as to simulate planned solutions. Surrogate measures are most often used in research conducted using microsimulation traffic models and driving simulators.

### 2.2. Driving Simulator Studies

Another method of data collection that enables the simulation of the real road environment is the use of a driving simulator. Driving simulators aim to reproduce the real road environment in a virtual world by placing participants in a mock-up of the vehicle interior and displaying the moving road and its surroundings on the screens. The most advanced simulators are high-level ones, which use virtual projection on screens around the vehicle and a mobile base platform on which the vehicle is placed [62]. The advantages of driving simulator research over field research are as follows [45]:

- possibility of using proactive research methods,
- generally unlimited possibility of defining the road environment according to the criteria assumed by the researcher,
- high level of detail and scope of collected data,
- ensuring the safety of test participants even for tests that would be dangerous in the real environment.

The main disadvantage of driving simulator tests is the limitation of visual realism that can be offered [44]. Due to limited realism, which can contribute to abnormal driver behaviour, the validity of driving simulator tests is quite often questioned [44,62]. However, many studies proved that driving simulators tend to achieve a high level of relative validity [45,46,47] and can be an important tool for comparing safety aspects between different controlled experimental scenarios.

Similar or SSM-related measures were considered for research using driving simulators. For example, one of the safety measures is Time to Line Crossing (TLC). This is a measure used to determine the remaining time to collision in a conflicting situation before the vehicle crosses the lane border. One of the measures of driver distraction may be the so-called information on staying within a lane (lane-keeping), i.e., the distance between the longitudinal axis of the vehicle and the lane axis [63,64]. It is possible, then, to analyse the driver’s effectiveness of staying within a lane (lateral control capability), which in real conditions is usually assessed by measuring lateral acceleration and the Standard Deviation of Lateral Position (SDLP) [65]. SDLP is a measure similar to TLC, reflecting the degree of control the driver has over the vehicle in each particular driving situation and is related to the probability of going off the road. It should be emphasised that inadequate lane-keeping is one of the basic factors contributing to road collisions [66].

Blaschke et al. [67] stated that drivers who were given additional information via In-Vehicle Information Systems (IVISs) increased, in most cases, the distance between the vehicle’s axis and the lane axis (so-called lateral deviation). The tests were conducted in real conditions. The authors defined distraction as “any activity that diverts the driver’s attention away from the task of driving.” They also referred to the research presented by Klauer et al. [68] regarding the scope of additional activities performed by drivers (not directly related to driving a vehicle) and the likelihood of a collision in the case of these activities. In almost 80% of collisions and 65% of incidents (situations close to collision), the driver did not pay appropriate attention.

Peng et al. [65] studied the driving paths of 24 vehicle drivers, driving a vehicle under real conditions. Based on this, they were divided into two groups, i.e., drivers who watched the road in front of the vehicle and a group of drivers who directed their eyes off the road, performing an additional task. A comparison of the results of lateral deviation measurements between groups showed an increase in the standard deviation of the lateral deviation in the case of the group of drivers taking their eyes off the road. An increase in cognitive load can, in some special situations, result in increased effectiveness in lane-keeping. Such a phenomenon can be encountered, for example, in the case of icy roads, when drivers focus their efforts on the activities related to observing the surroundings and the vehicle’s behaviour to maintain control, ensuring the maintenance of the driving path and vehicle’s speed. A similar principle is described by He et al. [69]. The tests were performed using a high-level driving simulator. Drivers drove the vehicle in a crosswind, being tasked with maintaining a stable lane position while the speed was stabilised using cruise control. This enabled drivers to focus on keeping the vehicle within the lane. During this engaging task, drivers heard audio recordings of numbers spoken by a sound synthesising program. It was a so-called n-back task. Drivers performed an additional task of repeating the four numbers heard in the order in which they heard them. Then the difficulty level of this task increased and they had to repeat the numbers they heard in ascending order, becoming more involved in the task. The authors of the article concluded that in the case under study, the increase in cognitive load, which, although it disrupts the activities performed by the driver, increases the effectiveness of lane-keeping. The measures described above are useful for comparative driving simulator studies but are also difficult to determine in field measurements without the use of advanced vehicle equipment [30].

### 2.3. Application of Sensors to Improve Road Traffic Safety and SSM-Related Microsimulation Studies

The traffic management tasks, including road traffic safety management, cover three main areas, which are:

- estimation of the traffic state in which data from different traffic sensors and traffic flow models fed by them are used to reproduce the traffic state picture of the whole road network (e.g., in terms of traffic density, speed and current dynamics of changes in traffic parameter values),
- prediction of the traffic state in which traffic projection in the future is calculated (short-term predictions are used to address traffic control issues),
- optimisation of traffic control measures (e.g., algorithms such as route guidance, VSLs, ramp metering, incident detection, etc.), the results of which are transmitted to the traffic control systems using actuators (traffic signals, VMSs, other roadside or in-vehicle information panels, etc.), including emergency events when traffic incident management is activated.

The use of the appropriate type of sensors enables us to collect the necessary and adequate data for a given traffic management process, but also data for modelling traffic control systems using simulation methods to improve traffic management strategies and tasks. Over recent decades, sensor technology has been developing more and more rapidly and has become ubiquitous. This has opened up new opportunities for the establishment and development of ITS services and the use of data for traffic modelling, including road safety studies. Sensors improving traffic safety can be installed both in vehicles and in the road environment. In the case of in-vehicle systems, speed sensors, RADAR and laser beams, micro-mechanical oscillators, cameras, inertial sensors, proximity sensors, ultrasonic sensors and haptic and night vision sensors are most often used to improve traffic safety. They are part of safety systems that focus on near real-time recognition of accident hazards and events [70,71,72,73]. The behaviour of drivers changes while warnings from sensors occur. As the number of such vehicles increases, this will need to be taken into account in the modelling of drivers’ behaviour, including the modelling and estimating of surrogate safety measures. Sensors that can be used to determine the distance between a vehicle and another one can be, for example, ultrasonic or electromagnetic sensors. Ultrasonic sensors allow for the identification of the distance between a vehicle and an object, warning the driver when he or she is approaching another vehicle above a defined distance threshold. Electromagnetic sensors are used to warn the driver when another vehicle is in an electromagnetic field generated around the bumpers. These types of sensors can be used to calculate the number of traffic conflicts not only between vehicles but also between vehicles and other objects in the road environment. The disadvantage of this type of sensor is a reduction in measuring accuracy due to humidity and temperature. Speed sensors and RADAR sensors are used to warn the driver of potential danger when changing lanes or detecting movement out of the lane [74]. Information from these sensors can also be used as a basis for analysing surrogate safety measures. Accelerometric and gyroscope sensors are used in conjunction with Global Positioning Systems (GPS) to improve the accuracy of navigation systems for determining vehicle parameters such as position and speed. The accuracy of the data obtained from such solutions does not appear to be sufficient to model traffic at the microscopic level and thus to estimate SSMs. Light Detection And Ranging (LIDAR ) enables vehicles (especially autonomous vehicles for which it is one of the key elements) to observe the road environment through 360° continuous visibility and very accurate depth information. LIDAR was applied for the collection of surrogate safety measures [75]. Sensor data (timestamps, the precise location of other vehicles and objects) can be used to estimate and validate SSMs. Camera-based image processing methods are used in in-vehicle systems to monitor the position of the driver’s head and eye activity. It enables the detection of fatigue, unusual vehicle behaviour (lane departure) [76], the appearance of an object within the road (e.g., a sudden pedestrian or an animal crossing the road, the appearance of another object on the road) and is also used as a basis for night vision applications. Object appearance around the vehicle recorded by the camera system can be useful in estimating SSMs. Other sensors that can be used in SSM estimation are Radio Detection And Ranging (RADAR) and laser sensors. They constantly scan the road in the vicinity of the vehicle to detect dangerous proximity to other vehicles or objects. Dangerous proximity detection allows safety applications to adjust the throttle and apply the brakes to prevent potential collisions. The RADAR sensors use radio waves to determine the distance to an obstacle. Applications notify the driver when a hazard is detected and can automatically apply the brakes to avoid a collision [70].

Mobile system data [77] (connected vehicles, Internet of Things, smartphones, new ways of information flow and cloud computing) enable the low-cost determination of vehicle speeds [78], vehicle travel time [79], vehicle tracking profiles (instantaneous speed, acceleration, deceleration) [80] and road safety performance assessment [81]. Data from mobile systems (especially vehicle tracking data) can be a valuable source of data for calibrating traffic models and estimating SSMs (if they are collected continuously) [80,82]. Further limitations on the use of individual sensors and the possibilities to use them for estimating SSMs are discussed below in the section on sensors installed in the road environment.

The automotive industry has invested a lot of funds in increasing safety, performance and comfort in vehicles by using sensors. However, the collection of traffic data with sensors along the roadside remains one of the main challenges for the development of ITS services. The deployment of sensors in the transport network provides drivers with many services, such as traffic management in road networks (traffic control, speed control, accessibility management, detection of incidents and objects on the road). Appropriate control strategies aim at improving the safety, reliability and resilience of the road network. Sensors can be divided into two categories (invasive and non-invasive), depending on their location in the road environment [83]. The invasive sensors are installed on the roadway surface. They are characterised by high accuracy, but also by relatively moderate installation and maintenance costs (installation and maintenance often require the temporary closure of road lanes and can contribute to shortening the life cycle of the pavement). Two groups of the invasive sensors are most commonly used: passive magnetic sensors and inductive loops, which send data to processing units. Inductive loops are most often used on Polish roads. The main advantage of invasive road sensors (especially inductive loops) is their technological maturity and large experience base. They have been widely implemented and are characterised by high accuracy in the detection of basic traffic parameters (volume, presence, occupancy, speed, headway, gap). These sensors are also insensitive to inclement weather (rain, fog, snow). The accuracy of the data collected by these sensors enables their use in traffic modelling and SSM estimation. The disadvantage that is described later in this paper is the possibility to collect data only in road cross sections where the sensors are installed. Alternatives to invasive sensors are non-invasive technologies [84,85].

The most promising non-invasive sensors that can be used in studies based on SSMs are RADAR sensors and a Video Image Processor (VIP). RADAR sensors emit low-energy microwave radiation, which is reflected by all objects in the detection area. We can distinguish between different types of RADAR sensor systems. The first type is Doppler systems, which allow for counting the number of vehicles and their speed. There are also continuous-wave RADAR with frequency modulation, which are used to measure the traffic volume, speed and presence of vehicles. RADAR sensors are very accurate and easy to install. They support multiple lane operation and can operate in the dark or adverse weather conditions. Their main disadvantage is their susceptibility to electromagnetic interference. In a Video Image Processor (VIP), video cameras placed on the roadside collect and analyse the video images from the road section or intersection using advanced software to determine changes between successive image frames. This technology enables the measurement of traffic parameters such as traffic volumes, speed, presence of vehicles and classification of vehicles. The main disadvantage of VIP systems is that they are prone to performance degradation due to adverse weather conditions (rain, fog, snow, wind) or vehicle shadows, occlusion and vehicle/road contrast [86,87]. A VIP was applied for the collection of surrogate safety measures [88]. The use of sophisticated algorithms in the software enables the analysis of the trajectory of individual vehicles on a given road section, which makes RADAR and VIP sensors the most suitable sensors for traffic safety analysis with SSMs.

Other types of sensors that are mainly used in road cross sections can also be mentioned: infrared, acoustic array and ultrasonic sensors. These sensors allow us to measure traffic parameters and support multiple lane operation. Sensors (e.g., piezoelectric, quartz, tensometric, fibre optic or capacitive) are also used to weigh vehicles in motion. These sensors can also be used to count the number of vehicles, their speed and to determine their classification, but due to high installation and maintenance costs, their main goal is to weigh vehicles as a part of Weight-In-Motion (WIM) systems. The use of WIM systems improves traffic safety. The risk of an overloaded truck driver being involved in an accident is higher than with a legally loaded truck. Moreover, the involvement of overweight vehicles in road accidents increases the severity of accidents [89]. Video image processing techniques and RADAR sensors (or sets of different sensors including image capture connected to a traffic signal controller) are often used to detect or predict traffic violations. For instance, when a vehicle exceeds the speed limit, as well as when a vehicle crosses the stop line at a junction or at a pedestrian crossing when red signals are displayed [90,91]. Information on the scale of the violations may be taken into account when assessing the safety of selected elements of the road system. The data collected by the sensors described above may complement the standard traffic modelling data. However, these are most often data collected at specific points on the road without recording the trajectory of vehicles and changes in the dynamics of their movement on the road section. The usefulness of such data in traffic modelling for SSM analysis along road sections is limited.

Roadside sensors are used in traffic safety management to collect data on the travel time of vehicles on a given road section. Automatic Number Plate Recognition (ANPR) cameras or Bluetooth and Wi-Fi scanners located at the beginning and the end of a road section enable the identification of an individual vehicle (in the case of ANPR cameras by the vehicle registration number, in the case of Bluetooth/Wi-Fi scanners by the Media Access Control (MAC) address—MAC number of the electronic device) and calculation of its travel time based on recorded time stamps. The main element of the data processing module is algorithms analysing the collected data to detect incidents on the road section (different techniques are applied to deal with erroneous or missing values, e.g., time series analysis, Kalman and particle filtering, neural networks, fuzzy logic). The incident detection algorithm searches for changes in the length of travel time between measuring points. In case of a sudden and unjustified change, a notification is sent to the traffic management system. The immediate detection of an incident results in a reduction in the time needed for emergency services to help the victim and the early activation of traffic control strategies (warnings displayed on the VMSs about the incident occurrence, detours, variable speed limits or road closures) to reduce traffic disruptions. A study based on the simulation of reactive VSL systems shows that the accuracy of information from the sensor stations, prediction of traffic conditions, estimation of time and place of the incident and the extent of the impact of the incident on traffic conditions are essential for the performance of a VSL system [92]. The data collected by the ANPR cameras and Bluetooth/Wi-Fi sensors can be used to calibrate and validate macroscopic, mesoscopic and microscopic traffic models in terms of travel time on the road sections and also for the calibration of time-dependent Origin–Destination (OD) travel matrices [93]. Incident detection is also possible by using video image processing methods or monitoring volume, speed and occupancy variations. It can be done by sensors located upstream and downstream of the incident (traffic measurement stations including inductive loops are most often used for this purpose) [94]. Data collected from sensors may also contain information about other road network disturbances (weather and state of pavement conditions, unexpected demands). Such data, if available, may be used for traffic modelling in the circumstances of a traffic incident occurrence.

Nowadays, an intensified development of Cooperative Intelligent Transportation System (C-ITS) services is observed. C-ITS services enable information exchange between vehicles (Vehicle To Vehicle—V2V) or vehicle and infrastructure (Vehicle To Infrastructure—V2I). Operating transport management systems are mostly not prepared to use Floating Car Data (FCD) or exchange data with vehicles. It is necessary to indicate the direction of development of these systems and to verify their architecture. Technological developments are therefore giving rise to integrated data sources that should be able to be used in research. The challenge is to process big data and merge them to use them in traffic modelling and safety assessment using SSMs. Nowadays, sensor data in vehicles and VIP and RADAR data are the best solution for traffic modelling and vehicle trajectory studies in the SSAM. Further development of methods based on the fusion of data from sensors located in the road environment and FCD data is required [95,96]. ANPR and Bluetooth/Wi-Fi sensor data are useful for travel distribution modelling and traffic model calibration or validation and can be complemented or replaced by mobile phones or electronic devices in vehicle location data.

Driving simulators are useful research tools in case of difficulties in obtaining data from mobile sensors or sensors located in the road environment. High-end simulators enable the recording of about 60 parameters related to the simulation, the location of the vehicle and its control mechanisms, and the quantities characterising the vehicle’s interaction with the environment. The simulator is a realistic simulation environment allowing for driver behaviour assessment in terms of road safety. Therefore, it is a laboratory research tool constituting a multisensory stand-in for a drive [97]. It enables directly recording parameters such as the steering angle and the degree of brake and acceleration pedal pressing. It also enables the recording of values necessary to calculate parameters such as the distance to the vehicle in front and the vehicle position vector in three dimensions with a timestamp. Another calculated parameter used to measure drivers distraction is vehicle position (lane-keeping). Simulators are useful in determining the dynamics of speed changes along the road and can support traffic modelling and SSM estimation.

Sensors play a key role in the collection of data for the improvement of services related to road traffic safety as well as data necessary for scientific research supporting the development of ITS services. It is important to make use of the fusion of data from many available sources (including sensors located in the vehicle and the road environment as well as mobile devices or systems) [98,99,100,101,102].

It is, therefore, reasonable to ask how the driver will react when further information is displayed on variable message signs, and the driver is involved in the analysis of the presented content. Then, as in the experiment presented in [67], will the lateral deviation increase? Will the level of traffic safety measured with SSMs decrease because of this? What will be the impact of speed harmonisation and speed reduction recommendations on traffic safety? This paper presents the possibilities of using data from various sensors to develop simulation models, which allowed for an attempt to answer the above research questions.

## 3. Methodology and Selected Results of Research

The methodology and results of research presented in Section 3 are based on the use of research tools such as traffic models (macroscopic, mesoscopic and microscopic) and driving simulators. Section 3.1 presents the process of developing test road network models and travel models that are developed based on real data. Moreover, the characteristics of the mesoscopic dynamic traffic model and the process of calibration of the microscopic model (including the calibration of the car-following model) using data from the traffic measurement stations are presented. Scenarios studied in the RID-4D project are also described, as well as scenarios that are used as a basis for the development of VSL models. Section 3.2 presents the process of tests using a driving simulator. The results of the research are used to further calibrate the microscopic traffic model in terms of drivers’ behaviour (changes in speed and dynamics of these changes) along the road section where VMSs are located. In the studies conducted with the use of a driving simulator, the influence of information displayed on the VMSs on lane-keeping by the driver is additionally identified. Section 3.3.1 presents the results of studies on the impact of VSL application (taking into account the location of VMSs) on road safety and traffic conditions. The research was conducted using a microscopic traffic model and the SSAM. In Section 3.3.2 the studies of additional scenarios of VSL impact are presented. Scenarios assumed the occurrence of different types of incidents in the road section (taking into account different incident duration and the scale of capacity limitation of the major road).

### 3.1. Development and Calibration of the Microscopic Test Models

The studies using microscopic models were part of research in which a multilevel approach was applied [9,103]. In the multilevel approach, macroscopic models (PTV VISUM software) [104] were used at the first stage of studies to obtain typical traffic distribution data in the road network with the use of the National Traffic Model. Detailed research on the influence of selected ITS service implementation on the road safety and traffic efficiency was conducted using mesoscopic (SATURN software) [105] and microscopic models (PTV VISSIM) [106]. Moreover, in the first stage of the study, available raw data from traffic measurement stations on motorways and expressways were collected. Inductive loops are the main elements of traffic measurement stations. Loops are located in selected road cross sections on each lane (in Poland, in the case of rural roads, mainly on motorways and expressways). The layout of two loops one after another on each lane enables measuring instantaneous speed and the classification of vehicles based on their length. Data from the traffic measurement stations provided an essential basis for the calibration and validation of macroscopic (traffic volumes, vehicle classification, average speeds), mesoscopic (traffic volumes, average speeds, vehicle classification) and microscopic (traffic volumes, speed values, vehicle classification, time headways between vehicles) models. The large number of available data allowed for the selection of a data set for model calibration and a control data set for model validation (including test network models and control models of the real road network). Models of the real road network were used to validate the adopted methodology in terms of traffic conditions on the road network in the case of typical traffic conditions and the circumstances in which the traffic incident occurred.

ViaToll system data were also collected to determine the routes of vehicles. The primary task of the ViaToll system is electronic toll collection on national roads. Vehicles with a maximum permissible weight of more than 3.5 tonnes and buses regardless of the maximum permissible weight are subject to payment. Other vehicles can voluntarily join the system and pay the charge on toll motorways. The basic elements of the ViaToll system are devices equipped with Dedicated Short-Range Communication (DSRC) readers, which recognise the passing vehicles. Some of the gantries are equipped with laser sensors or ANPR cameras and they collect data on all passing vehicles. Other gantries allow data collection only on vehicles with an on-board unit (ViaBox for heavy vehicles and ViaAuto for other vehicles). The system classifies vehicles into eight categories. The system does not measure vehicle speed. Data from these measurement points are aggregated to an hourly interval, for each lane. There are currently more than 700 gantries with DSRC readers on national roads. DSRC operates based on separate short-range radio communication in the 5.8 GHz band. Data from 30 stations equipped with laser readers were used to calibrate the National Traffic Model (macroscopic model). ViaToll data were also used to determine the percentage of vehicles that were leaving the motorway or expressway after the incident occurrence (depending on the length of the incident). In the dynamic mesoscopic model, the process of route selection by drivers in subsequent periods of the incident was reproduced, taking into account the traffic volume.

In the next stage, the topology of the road network in the corridors of Polish motorways or expressways was analysed. The analysis took into account the structure and traffic alignment within road interchanges, distances between nodes and characteristics of alternative routes located in the major road corridor. Based on the collected data and the research carried out, test models of the road network were developed for selected road classes (expressways: S 2/2 and motorways: A 2/2, A 2/3) [34]. According to the Polish road classification, both motorways and expressways are designed and built for international or national motor traffic over longer distances. These roads are intended exclusively for motor vehicle traffic. The main difference between the discussed road classes is the maximum permitted speed (140 km/h on motorways and 120 km/h on dual carriageway expressways). Different permitted speeds and related design speeds determine the differences in the geometric parameters of the roads. These include, among others, the width of lanes, horizontal and vertical curves, roadway inclinations, width and presence of the emergency stopping lane, geometric solutions and parameters within road interchanges. The second important difference between motorways and expressways is their accessibility. The minimum permitted distance between interchanges on motorways is 15 km (within large cities, 5 km) and on expressways, 5 km (within cities, 3 km). In the test network models, the calculated average distance between interchanges along major roads based on the actual road network topology for the A 2/2 motorway was 15 km and for the S 2/2 expressway, 10 km. The length of roads in the test network for the A 2/2 motorway corridor was about 120 km, including 45 km of the major road, while in the S 2/2 expressway corridor, it was 74 km, including 30 km of the major road.

**Figure 1:**
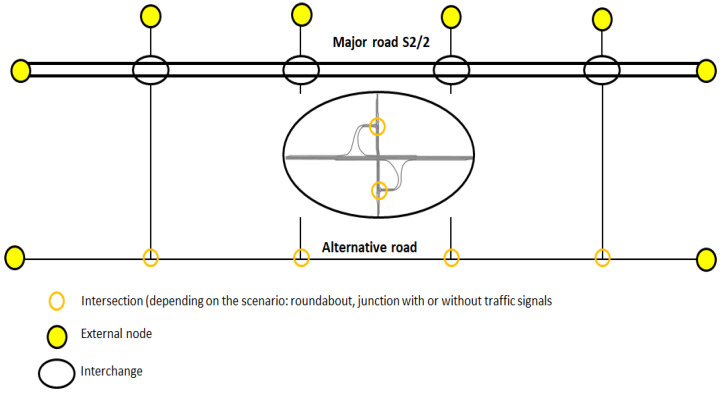
Test network for S 2/2 expressway corridor.

Traffic simulations with the use of the test network made it possible to conduct studies on the impact of the location of VMSs with speed limits displayed on them on the level of road traffic efficiency and safety. An example of a test network model for the S 2/2 expressway is shown in Figure 1 [34].

Selected scenarios of the road network and traffic intensity were analysed, taking into account the occurrence of incidents on the major road to determine the impact of the use of ITS services on road safety and traffic efficiency. In addition to the road network topology, different types of interchanges and junctions along the major road and alternative routes were considered and defined in the simulated scenarios. The occurrence of an incident on the road resulting in blocking one or two lanes on the main road was assumed during the development of test models for selected scenarios. The analyses also took into account cases where the incident did not cause lane blocking. A reduction in road capacity during a simulated incident was adopted in mesoscopic models and, in the next step, in microscopic models. In addition to capacity changes due to traffic distribution, the rubbernecking phenomenon was taken into account in capacity limitation based on research [107,108]. The results of traffic assignment in mesoscopic test road networks were used to develop microscopic models.

Mesoscopic models of the test road network allowed us to proceed with traffic assignment, taking into account variable traffic conditions and capacity limitation resulting from queues, delays and the stopping of vehicles in traffic flow at junctions, road interchanges and individual road sections. A quasi-dynamic model was applied during the stochastic traffic assignment process to obtain more reliable results. Over-capacity queues were moved to subsequent defined periods by using the model (the simulations were divided into 30-min periods). The application of a quasi-dynamic model allowed us to take into account the dynamics of changes in traffic conditions in the face of the temporary blocking of the road by incident occurrence [9]. The mesoscopic model was used mainly to analyse the impact of incident management, while the microscopic model (fed by the traffic distribution in the road network and traffic volume data from the mesoscopic model) was used for the studies of Intelligent Transport System (ITS) services. This included scenarios involving providing the drivers with information through content displayed on VMSs. The selected scenarios were defined based on Regional Fire Departments’ databases with data on the duration, location and type of incidents. The share of vehicles choosing an alternative route in the periods when incidents occurred was estimated based on the data from the ViaToll system. The data from the ViaToll system enabled determining the routes of the vehicles in selected areas of the road network under conditions of different incident duration compared to the traffic distribution in the network under non-incidental conditions. It was done based on the identification of the vehicles and the time stamp of their appearance at subsequent ViaToll measurement points. The collected data were used to calibrate and validate the test network models. Independently, mesoscopic and microscopic models of selected corridors of the real road network (sections of A1 motorway and S6 expressway corridors) were developed. The comparison of real and model results allowed for a positive validation of the adopted models and the methodology of their development (traffic distribution in the network under the conditions of incident occurrence, traffic volume values and speed on selected sections of the network were compared). The data on the duration of incidents, their location and the scale of disturbances (closing of the entire roadway, the closing of one lane, etc.) were obtained from the reports of the Regional Fire Departments, which complemented the data collected by the ViaToll system and traffic measurement stations.

The representative hourly traffic volume in the different scenarios was classified into cohorts (Table 1) [9]. The classification of cohorts was made based on traffic data collected by the traffic measurement stations. The large amount of data collected by the sensors and data variations in terms of traffic volume values and circumstances (typical states, conditions with the occurrence of an incident) allowed us to develop traffic models for various conditions in the road network. The purpose of adopting cohort sets was to determine the frequency of occurrence of selected types of incidents causing different traffic limitations (number of lanes blocked) on particular road classes during the total road operation.

The calibration of the microscopic models was carried out, taking into account time headways between vehicles and vehicle speed distribution. The data from traffic measurement stations (separately from selected expressways and motorways) and data obtained from tests with a driving simulator were used for calibration. Raw data (vehicle after vehicle) obtained from the traffic measurement station were used to determine the probability distribution (empirical cumulative distribution functions) of the choice of speed and time headways by drivers of different classes of vehicles (passenger cars and delivery vans as well as HGVs and buses).

**Figure 2:**
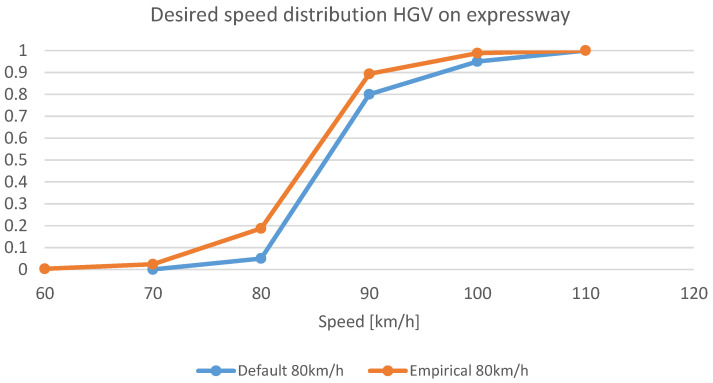
Comparison of the default VISSIM desired speed distribution and the empirical distribution for heavy goods vehicles (HGVs) and buses on the expressway.

The speed at which the driver is not influenced by other road users is defined as the desired speed. Desired speed was a variable used to calibrate the microscopic model. Traffic conflicts may occur when a vehicle interacts with slower-moving or queued vehicles during sudden braking or lane changing. The determination of speed and time headway distribution was developed based on data from traffic measurement stations for randomly selected days from different seasons. A comparison of the empirical desired speed distribution with the default VISSIM distribution for heavy vehicles and buses on the expressway is shown in Figure 2. The basic assumption is that for vehicles travelling slower than the displayed limit, the speed limit on VMSs will not affect these vehicles. The actual impact of VMS devices will be visible for vehicles travelling faster than the permitted speed. Information on the variable sign for what reason the speed is limited makes drivers more willing to adapt to it. Desired speed distribution functions were modified in such a way that the percentage below the speed on the VMS is identical to the initial one. On the other hand, the modification above the value indicated by the VMS included achieving speed values similar to those observed in reality, e.g., quantile 85 for a speed 20 km/h higher than allowed. Based on this, the distribution functions for the 80 km/h and 100 km/h limits were developed and implemented in the model. The modelling of the speed management service was performed using COMInterface. It is one of the VISSIM modules that allows the user to develop scripts that execute commands during the simulation that affect model elements and driver behaviour. An algorithm was developed to modify speed limit values on VMSs on the major road. During the simulation, the algorithm collected traffic volume data from the virtual sensors located in VMS areas and decided to change the speed limit every 5 min.

Virtual sensors correspond to inductive loops or other sensors detecting vehicle appearance and collecting basic traffic data. Virtual sensors performed two tasks in simulation models. The first task was to start the traffic control algorithm (displaying the corresponding speed limit on the VMS) in case of exceeding the defined traffic volume threshold. The second task of the virtual sensors was to monitor indicators of model calibration and validation (traffic volumes, vehicle speed values, time headways). An important element was the location of the virtual sensors in the test network model. Placing the virtual sensor too close to the VMS could cause the algorithm to indicate the conditions for changing the displayed speed with a delay. Such a situation may affect drivers’ behaviour in terms of speed and distance to the next vehicle. The location of sensors is one of the key issues and should be thoroughly studied in future research work.

For the study, the following boundary conditions were adopted to change the speed limit value on VMSs on the expressway:

- 120 km/h ≤ 1000 veh/h/lane,
- 100 km/h > 1000 veh/h/lane,
- 80 km/h > 1550 veh/h/lane (if the VMS series was used, a speed limit of 100 km/h was displayed on the first VMS after the interchange and a limit of 80 km/h on the subsequent VMS. If one sign was located on the road section between the interchanges, a speed limit of 100 km/h was displayed on it, which followed the applicable regulations).

**Figure 3:**
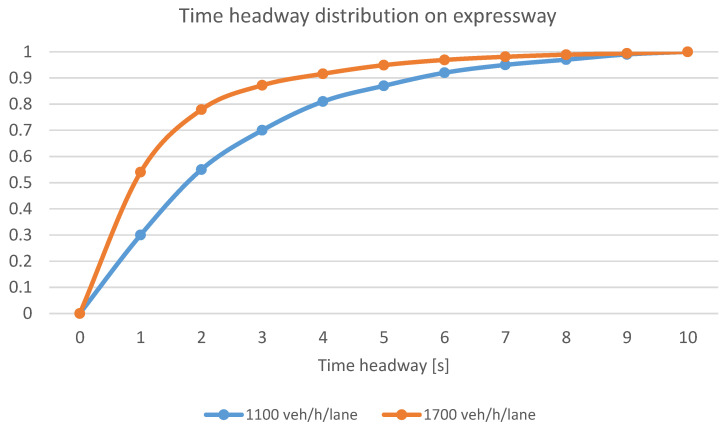
Time headway distribution on an expressway for 1010 veh/h/lane and 1700 veh/h/lane.

Another variable that was used for the model calibration was following time headway. This variable largely reflects driver behaviour, affecting road safety and capacity. The presence of a too short time headway between vehicles increases the risk of an incident in case of specific driver behaviour. Time headway is one of the variables in the Wiedemann 99 car-following model [106]. Researchers usually calibrate the models by setting a single variable value, resulting in a less realistic simulation of drivers’ behaviour [109]. Time headway empirical data with a duration of less than 10 s (the limit above which free-flow traffic conditions occur) were used to develop cumulative distribution functions and to calibrate test models for representative traffic volumes in each scenario. Selected time headway cumulative distribution functions for different traffic volumes are shown in Figure 3 [34].

In the process of the calibration and validation of the model, data sets were used which were extracted for particular road classes and traffic volumes that were representative of the cohorts presented in Table 1. Data on the speed of the vehicles and the time headways between them were selected for each representative value of the traffic intensity (Table 1). The validation process used control data sets from different periods or other traffic measurement stations than the data used in the calibration process. The model validation related only to the solutions presented in the baseline scenario (without a VSL system) due to difficulties in finding a test field on Polish roads (no solutions with operating VSL systems or no data in the vicinity of the operating of a single VMS).

In the first step, the functions of the time headway distribution for individual road classes and representative traffic volumes in ten 1-s duration intervals (ranging from 0 to 10 s) were defined. In the validation process, the distributions of the sample frequencies over the intervals for the data set to develop the traffic model and control data set (percentage of the number of vehicles in each interval) were compared. No differences between the distributions of more than 12% (in most cases up to 8%) were noted for the individual intervals, which was considered a satisfactory result. The resulting cumulative distribution functions contributed to the simulation traffic models in the calibration process and were not subject to further changes.

Traffic models have been developed for representative traffic intensity values (Table 1). During the calibration process, it was assumed that the traffic intensity values from the models would not differ by more than 6% when concerning the representative values. The above requirement was fulfilled.

Before starting to calibrate the model, the speed distribution functions for the different road classes, representative traffic volumes and vehicle categories were defined at 10 km/h intervals (in the range of up to 150 km/h). During the calibration process, the distributions of sample frequencies in individual intervals for the data set to develop the traffic model and the results from the model (percentage of the number of vehicles in each interval) were compared. No differences between the distributions of more than 16% (most often up to 13%) were observed for individual intervals. The most significant differences were observed in the speed intervals above 90 km/h, and in the other intervals for higher traffic volumes. The Mean Absolute Percentage Error (MAPE) for mean speeds in road cross sections calculated from observed data and the modelling results did not exceed 6% for passenger vehicles and 4% for heavy goods vehicles (values from the upper error range were observed for higher traffic volumes). The results were found to be satisfactory.

The validation process compared the distributions of sample frequencies in individual speed intervals for the model data set and the control observed data set. No differences in distributions exceeding 27% (most often up to 18%) were observed for individual intervals. The most significant differences were observed in the speed intervals above 80 km/h. The MAPE for the mean speed in road cross sections calculated from the observed data control group and the model results did not exceed 12% for passenger vehicles and 6% for heavy goods vehicles (values from the upper error range were observed for higher traffic volumes).

The results of driving simulator tests were used to calibrate models in VSL scenarios. Data enabled the identification of areas of speed change by drivers in response to a message on VMSs [97]. Due to the lack of field test sites, it was not possible to validate them. The conclusions of the paper indicate plans to carry out verification using data from field measurements. The capability of the driving simulator is limited in terms of triggering linear and angular accelerations and also due to imperfect movement patterns of the vehicle and its components (e.g., tyre/wheel model). The screen is located at a short distance from the driver’s eyes and focuses his or her eyes on a different point than when driving. Despite these disadvantages, driving simulators are commonly used as a testing tool, as they enable simulation of driving in repeatable, safe conditions. The screen is placed approximately 3 m from the driver’s eyes in the AS 1200-6 simulator to minimise its drawbacks. It was also necessary to calibrate it according to the perception of the driver. The AS 1200-6 driving simulator was calibrated according to the individual perceptions of professional drivers with extensive driving experience. Although it was not possible to validate the results from the driving simulator, the data used enabled the development of comparable simulation conditions in the simulation scenarios.

### 3.2. Driving Simulator Study Results

The results of studies carried out using a driving simulator were also used to calibrate the microscopic test model. The data obtained from the studies, with the use of the high-end AS 1200-6 driving simulator, made it possible to take into account the behaviour of road users and to calibrate the microscopic model in terms of changes in the speed of vehicles before and behind the VMS location [97].

In studies using the driving simulator, the research group consisted of 60 people. The conditions to participate in the studies were to have a valid driving licence and to drive at least 2000 km per year. Participants were divided into three age groups: 18–24, 25–49 and over 50. There were 20 persons in each age group. The set of research scenarios involved conducting a simulation of driving past a sign or a VMS.

**Figure 4:**
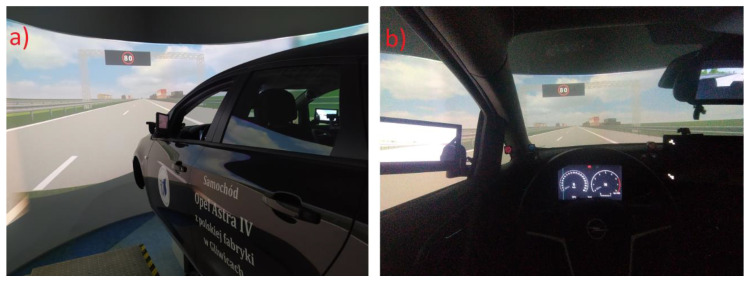
Simulated road environment (**a**) and high-level driving simulator (**b**).

As part of the selected scenarios, six gate support structures (so-called gantries) were placed, on which graphic symbols corresponding to the signs and the VMS, were presented (Figure 4). Speed limits, information on traffic incidents and information on the weather conditions and alternative routes, as well as instructions for the drivers (e.g., about the need for lane changes), were presented.

The purpose of the subsequent series of research experiments was to assess the impact of services providing information to the drivers on their behaviour. During the experiments, several dozen parameters related to the simulation and the vehicle itself moving in a virtual environment were recorded. One of these parameters was SDLP, mentioned earlier, as well as outputs on change of speed in the section before and behind the location of the VMS and the range of speed changes, taking into account the drivers’ different reactions to the information about the speed limit that was displayed (outputs were used in the process of microscopic traffic model calibration).

**Figure 5:**
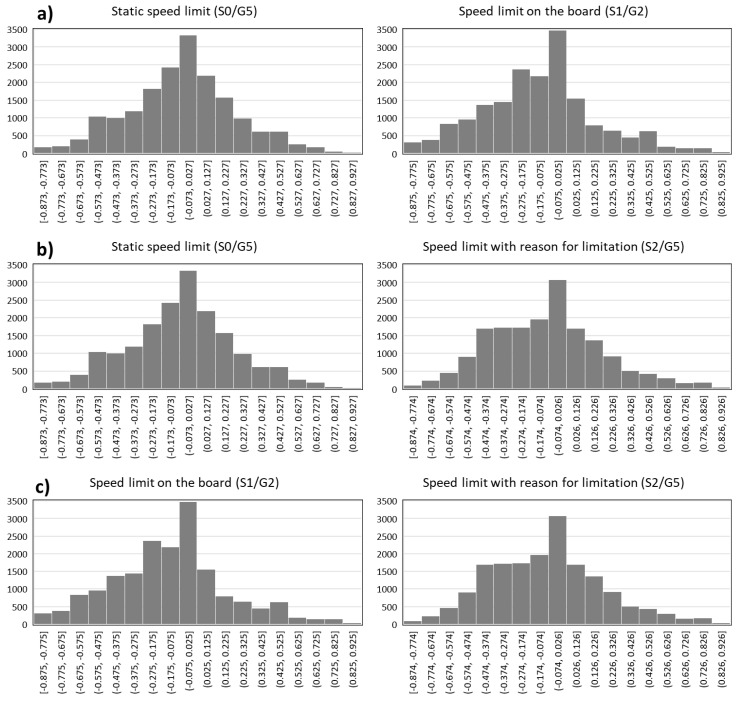
Comparison of pairs of the lane-keeping data under individual research scenarios.

The data were recorded for two weather conditions (precipitation and no precipitation), reflecting actual road conditions in Poland (the number of days with precipitation is approximately equal to the number of days without precipitation). Based on the deviation of the longitudinal axis of the vehicle from the lane axis (lateral deviation), one can draw conclusions about the additional load on the driver and the impact of the content presented on the value of this deviation. The lateral deviation is an objective measure of the driver’s efficiency and makes it possible to make conclusions about the distraction of the driver’s attention as a result of additional tasks [65]. Such a task may be observing signposting in the form of VMSs. Although they can be regarded as an element of the road infrastructure, and their observation as a driving activity, in the case analysed in this article, this marking was considered as an additional element of the road signposting, which may or may not have to be used as a tool to inform drivers about the traffic situation. Thanks to this, it is possible to assess their impact on lane-keeping, and thus on the road safety itself. To evaluate the impact of the information provided through ITS services, lane-keeping was compared in pairs over a distance of 400 m, i.e., 200 m before the gantry support structure and 200 m behind it. The comparison was made for the baseline scenarios (without the impact of ITS services) and with such services introduced. The comparison of pairs of lane-keeping data under individual research scenarios is shown in Figure 5. The figure shows the number of samples (the deviation of the longitudinal axis of the vehicle with a frequency of 50 Hz was recorded) in individual deviation intervals. Negative values in the given intervals indicate an increased deviation from the right edge of the lane. Scenarios with the introduction of a speed limit with the use of a road sign (S0/G5), VMSs (S1/G2) and VMSs with additional information about the cause of the limit, which was a slippery road surface (S2/G5), were considered.

For the cases presented in Figure 5, the average value of the distance between the longitudinal axis of the vehicle from the lane axis and standard deviation of this value was calculated (Table 2).

Comparing the effect on the driver of a static (S0/G5) and dynamic (S1/G2) speed limit (Figure 5a), an increase in the average lateral deviation was noted from 6.3 cm, in the case of a static sign, to 13.4 cm in the case of a VMS. The standard deviation also increased from 30.7 cm to 33.2 cm. A possible reason for this is the recent legal regulation of respecting the indications of variable road signs compared to traditional solutions, well known to drivers. This indicates that they are more responsive to traditional road signs and, in this case, the considered impact of ITS on lane-keeping did not bring the expected effect. However, the differences were negligible. Comparing the static speed limit (SO/G5) with the VMS (S2/G5) (Figure 5b) containing the reason for the speed limit displayed, it had a high impact on drivers’ speed. In the case of lane-keeping, the position deviation from the lane axis increased insignificantly from 6.3 cm to 9 cm and the standard deviation also slightly increased from 30.7 cm to 31.8 cm. Comparing the speed limit displayed on the VMS (S1/G2) with the same limit supplemented by the reason for the limit (S2/G5) (Figure 5c), an increase in lane-keeping was observed from 13.4 cm to 9 cm. The standard deviation decreased, from 33.2 to 31.8. This is an indication to apply solutions for imposing the speed limit, supplemented with information about the reason for the limit.

### 3.3. Microscopic Modelling Results

#### 3.3.1. Location of Variable Message Signs

The analysis of the state of the existing location of VMS devices on Polish motorways and expressways showed that the main arrangement is that with a single device between interchanges. Only on a few motorways (the A8 road is an example) are the devices located in series between interchanges. As part of the research, scenarios of VMS locations between interchanges were analysed. This paper presents the simulation results of three scenarios of VMS location on the expressway (S 2/2) with a traffic volume of 1700 vehicles per lane (the presented scenarios assume the use of junctions with traffic signals within interchanges on the major road and within the alternative route, as shown in Figure 1):

- W0—a baseline scenario—no service (VMS),
- W1—one VMS between interchanges,
- W2—VMSs between interchanges placed every two kilometres.

The impact of implementing speed control on road safety as measured by the number of traffic conflicts is presented in Table 3. The impact of introducing speed control on traffic efficiency measured by traffic conditions results from simulation scenarios on the test road network, including the major road and the alternative routes (expressway corridor), is presented in Table 4.

The Surrogate Safety Assessment Model (SSAM) was used to calculate the number and severity of traffic conflicts [51]. Two surrogate safety measures were used to assess the level of road safety in selected simulation scenarios: Time To Collision (TTC) and MaxDeltaV, which is determined by the speed difference between vehicles involved in a traffic conflict. Movement trajectories for every interacting vehicle were extracted from the simulation environment of microscopic models and compared to identify the occurrence of conflicts and their characteristics.

A traffic conflict should be defined as the occurrence of an interaction between two vehicles which would lead to a collision if one of the vehicles did not change speed or direction. TTC is defined as the period during which a collision would occur if neither of the two vehicles changed speed or the direction of driving [110]. The simulation results were used to compare different types of conflicts (lane change and rear-end conflicts on the sections of the major road between interchanges were taken into account). Equation (1) can be used to calculate TTC. A minimum assumed TTC value (TTCmin between 0.1 s and 1.5 s) was adopted to define the traffic conflict occurrence.

(1) $$TTC_{i}=\frac{X_{i-1}\left(t\right)-X_{i}\left(t\right)-l_{i}}{{\dot{X}}_{i}\left(t\right)-{\dot{X}}_{i-1}\left(t\right)}\;\;\;\;\;\;\forall {\dot{X}}_{i}\left(t\right)>{\dot{X}}_{i-1}\left(t\right)$$

where:

*X*—vehicle position,
$\dot{X}$—vehicle speed,
*i*—vehicle following the leader,
*i*−1—lead vehicle,
*t*—moment in time,
*l*—vehicle length.

The MaxDeltaV measure can be used indirectly to evaluate the severity of traffic conflict, which occurred when TTC was less than the minimum assumed value, i.e., when the conflict was identified. The relative severity of the traffic conflict can be determined by comparing the trajectories of two vehicles in terms of direction and speed at the moment when TTCmin occurs. The severity of the conflict is higher in the case of greater speed differences between vehicles involved in traffic conflict and an unfavourable angle of a potential collision. The angle at which the trajectories of vehicles involved in traffic conflict cross is a criterion for assigning a conflict type. The SSAM defines three types of traffic conflicts: crossing, lane change and rear-end. It is assumed that the conflict is identified as severe in the case of a difference in speed of vehicles exceeding 20 km/h [47].

It should be noted that in the analysed scenarios, saturated traffic conditions (1700 veh/h/lane) on the major road and in the remaining road network were assumed, which led to lower traffic speed. The results for all scenarios (Table 3) showed differences in the distribution of specific types of conflicts (2–3% crossing, 82–84% rear-end and 13–16% lane change) on specific parts of the test road network (39–53% on the major road between interchange areas, 39–50% at interchanges along the major road and 8–11% on the rest of test road network) depending on the scenario. In terms of the severity of conflicts assessed based on MaxDeltaV in the individual scenarios, the highest percentage of conflicts was observed on the major road and in the area of road interchanges. Due to speed limits on alternative routes, the percentage share of severe conflicts was marginal. Conflicts of the lane change type prevailed (50–61% depending on the variant) and rear-end (25–29%). In saturated traffic conditions, greater differences in speed between vehicles in the case of conflict were more frequent.

The effect of implementing variable message signs was most evident on the sections of expressways and motorways (this paper shows an example of research for an expressway with two lanes in each direction). The road sections studied did not include the area of interchanges with merging and weaving sections. As shown in Table 3, the most significant reduction in the number of conflicts that occurred in the W2 scenario (implementation of a VMS series along road sections). The reduction concerned both lane change conflicts (a decrease by 59% compared to the baseline scenario) and the most common rear-end conflicts (39%). The introduction of a service providing information about the recommended speed also resulted in a more significant reduction of conflict severity than in the case of their number. In the case of lane change conflicts, there was a reduction by 64%, for rear-end conflicts by 52%. The impact of speed reduction on the expressway sections was also noticeable within road interchanges. If a VMS was located closer to the interchange (in the case of scenario W2 with a series of variable message signs along the section), there was a reduction in the number of conflicts and their severity compared to the baseline scenario. Within the rest of the road network (alternative routes and routes connecting with the major road), there was a slight deterioration in the level of traffic safety measured in absolute values of the number of conflicts. In the whole road network under study, the introduction of the speed control service contributed to the reduction of the number of conflicts (by 24%) and their severity (by 38%).

**Figure 6:**
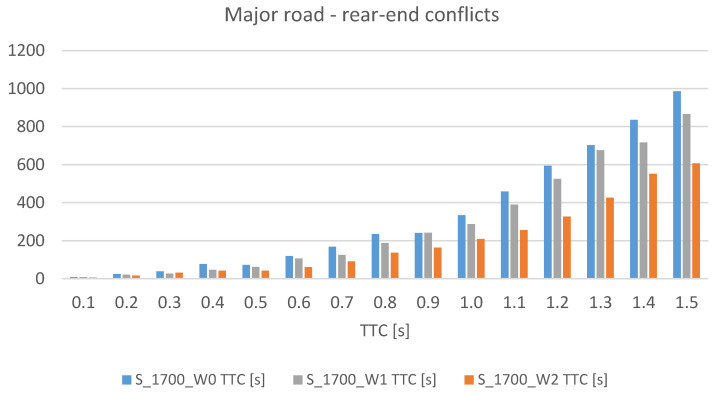
Distribution of occurrence frequencies of Time to Collision (TTC) values in individual scenarios.

The frequency of the TTC value occurrence in the scenarios of the localisation of variable message signs is presented in Figure 6. The figure shows an example of a TTC distribution for rear-end conflicts on sections of the major road. In scenarios involving providing the information on speed limits to the drivers, decreases in TTC were noted in all the considered intervals. The results showed a positive impact of the measures on the level of road safety. Conflicts for which TTC took values above 1 s were the most frequent. Similar distributions were obtained for lane change conflicts.

**Figure 7:**
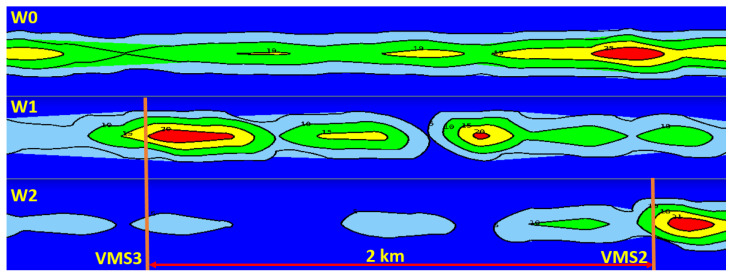
Areas of conflict concentration along the major road (the direction of traffic from left to right, conflict density represented by the size and the colour of the area, where a darker colour means a higher density).

**Figure 8:**
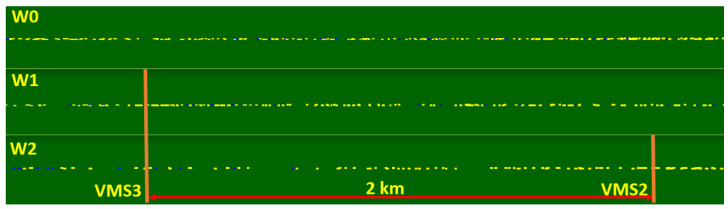
Distribution of individual conflicts along the major road section (yellow colour—rear-end type, blue colour—lane change type).

A useful tool in the study of the conflict occurrence process is the analysis of their concentration. In the case of the considered scenarios of variable message sign location, different patterns of conflict concentration areas were observed (Figure 7 and Figure 8). Figure 7 shows the areas of conflict concentration (conflict density represented by the size and the colour of the area, where a darker colour means a higher density). The distribution of individual conflicts along the major road section (rear-end type—yellow colour, lane change type—blue colour) is shown in Figure 8. For all scenarios considered, the first place where the conflicts accumulate is the first section of the major road, along the merging section at the place where traffic flows at different speeds: the point where entry traffic flow from the ramp and the expressway traffic flow meet. This contributes to the occurrence of lane change conflicts but also contributes to the formation of a shock wave, which is accompanied by rear-end conflicts and, in a smaller number, by lane change conflicts. In the absence of dynamic information about the speed limit (W0), conflicts arise evenly over the entire section between interchanges (Figure 8). The places where conflicts accumulate are irregular and, under saturated traffic conditions, are associated with the formation of short-term shock waves due to greater differences in vehicle speeds along the road section. The phenomena described above result in the highest number of conflicts and their highest severity (Table 3) among the scenarios under consideration. In a scenario where the use of a single VMS (W1) is taken into account, areas with an accumulation of conflicts appear at the place of impact of the VMS, in its vicinity (Figure 7), due to speed reduction by some drivers, which also contributes to the formation of shock waves. On the longer sections without VMSs, some drivers increase their speed, resulting in irregular areas of conflict accumulation similar to the W0 scenario (Figure 8). In the case of using variable speed limits with the use of a VMS series (W2 scenario), the concentration of conflicts was observed in the area of the first VMS on the road section directly behind the interchange (effect of merging traffic flows of different speeds) and before the second VMS in the series (Figure 7). In the remaining section of the road, a lower frequency of conflicts and fewer concentrated areas can be observed (Figure 7 and Figure 8), which proves the harmonisation of speed thanks to the use of a VMS.

The simulation results presented in Table 4 show an improvement of the traffic conditions in the whole test network due to the introduction of speed limit information and the resulting traffic harmonisation by placing more VMSs on a road section.

#### 3.3.2. Comparison of Scenarios Taking into Account the Occurrence of Incidents

Further comparisons took into account the lower level of traffic volume in the test road network and considered situations in which traffic incidents occurred. The research was carried out for the baseline scenario and scenario W2 assuming the location of a series of variable message signs in the section between interchanges (scenario W2 proved to be the most effective due to improved traffic conditions and road traffic safety). The traffic microsimulation for each scenario took 2.5 h.

This paper presents and compares the simulation results for two traffic intensity levels representing cohorts 1 (saturated traffic flow conditions) and 2 (free-flow traffic conditions) from Table 1. The results of simulations for the test road networks in the expressway corridor (S 2/2) are presented. Selected scenarios assume the occurrence of signal-controlled junctions within interchanges along the major road and on alternative routes.

The results of simulation scenarios assuming an incident in the major road middle section (scenarios W0b and W2b) are also presented. The simulated incident lasted 30 min and caused one lane to be blocked. The following are the characteristics of the selected scenarios in terms of incident occurrence and application of the ITS service, which informed drivers about the recommended speed via VMSs:

- W0—a baseline scenario—no ITS service (VMS),
- W2—VMSs between interchanges located every two kilometres,
- W0b—a baseline scenario—no ITS service (VMS), the occurrence of an incident,
- W2b—VMSs between interchanges located every two kilometres, the occurrence of an incident.

The impact of introducing speed control on traffic efficiency measured by traffic conditions from simulation scenarios on the test road network (Figure 1), including the major road and the alternative routes, is presented in Table 5.

The introduction of variable speed limits contributed to the improvement of traffic conditions, taking into account the whole road network located in the expressway corridor. The implementation of variable speed limits resulted in a decrease in total delays and the number of stops for both lower and higher traffic volumes. The improvement was more evident in the saturated flow conditions. Moreover, the occurrence of the incident resulted in a reduction of capacity on the major road. For this particular reason, the traffic assignment changed and congestion along alternative routes increased.

**Figure 9:**
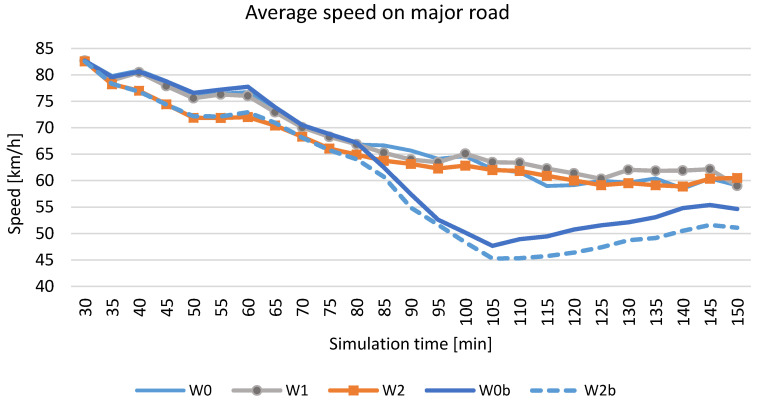
Average speed on the major road with traffic volume of 1700 veh/h/lane.

The introduction of ITS services related to speed control aims at improving the level of road safety by harmonising the speed of vehicles and reducing over-speeding both in normal traffic conditions and when an incident occurs. Traffic conditions deteriorated significantly both along the major road and alternative routes after an incident occurred in the road network. One of the main objectives of the implementation of ITS services is to reduce congestion and help to restore normal traffic conditions as quickly as possible after clearing a road lane blocked during an incident. Changes in average speed during the simulation in the selected scenarios of test models are shown in Figure 9 (scenario W1 with a single VMS on the road section between interchanges was included).

The occurrence of an incident between 60 and 90 min of simulation had a significant impact on the decrease in average speed in both the baseline scenario and the scenario with variable speed limit implementation. The average speed was lower in the scenario with the incident (W2b) compared to the baseline scenario (W0b). Still, the implementation of variable speed limits contributed to an increase in traffic volume along the major road to a value in the range near to its capacity, as well as improved the level of road safety (Table 6).

The results of the studies on the level of road safety using the SSAM are presented in Table 6. The presented results include the road network of the expressway corridor together with alternative routes. The positive impact of the variable speed limit on road safety is expressed in a reduction in the number of conflicts and their severity. It should be noted that there is a significant deterioration in the level of road safety in the case of increased traffic volumes in the studied road network. The implementation of the service providing information about speed limits on sections of expressways (excluding interchange areas) resulted in a reduction in the number of conflicts to 7% in the case of a lower traffic volume and to 24% in the case of saturated traffic conditions. The number of severe conflicts was also significantly reduced, to 16% and 38%, respectively. In the case of scenarios where an incident was assumed to occur, there was also a reduction in the number of conflicts and severe conflicts, but to a lesser extent than in non-incidental conditions. The results indicate a significant potential for introducing measures to harmonise vehicle speeds, resulting in an improved level of road safety.

## 4. Discussing the Results and Conclusions

One of the main objectives of intelligent transportation systems solutions is to improve road safety. The implemented solutions should be used to provide drivers with information and influence their behaviour in such a way that the above postulate can be realised. The relevant questions are, therefore, how to study the impact of ITS solutions on road safety and how sensors can be used within the presented research methodology. This paper presents measures that can be useful in road traffic safety research and can complement or replace traditional statistical studies, especially in the case of innovative implementations for which field or statistical studies are difficult to conduct due to their rarity. The key basis for the research is the sensors and data collected by the sensors, which made it possible to develop, calibrate and validate traffic models and then estimate surrogate safety measures. Surrogate safety measures enable us to study the effects of planned modifications to the existing ITS services and the search for optimal solutions to increase their effectiveness. Moreover, the research methodology presented in this paper allows for collecting data as a starting point for developing comprehensive models of ITS services’ impact on road efficiency and safety. It allowed us to obtain data to power the model, enabling the estimation of ITS services’ efficiency based on the Analytic Hierarchy Process (AHP) method and to estimate indicators for functional criteria for the AHP method [9,34]. There are no guidelines for introducing and designing VSL systems in Poland. There is also a lack of a method for assessing the effectiveness of the implemented solutions. The presented research methodology, which takes into account different levels of traffic intensity and occurrence of road incidents, may support the development of such guidelines. Field tests of the VSL service are also hampered due to the lack of testing sites in Poland. VSLs with a series of variable message signs have been used on some sections of motorways and expressways (e.g., on the A8 motorway or the S8 expressway). Usually, VMSs providing information about the recommended speed are installed on road sections between interchanges either singly (after passing an interchange) or in pairs (after passing the interchange and before the next). This practice makes it impossible to introduce an effective VSL system with a series of signs on road sections where this may be appropriate. The possibility of obtaining data on the periods when individual information is displayed on VMSs is very limited (such information is not collected in databases). Databases usually collect data from traffic measurement stations that are used to calibrate microscopic models. Traffic measurement stations, which include inductive loops, are most often used on Polish roads. Traffic measurement stations give the possibility to collect data only in selected points of the road system where the sensors are installed. Dynamic traffic safety management and traffic safety studies usually require the collection of traffic flow data from road sections. One of the ways to strengthen the detection systems and improve the quality of the collected data sets is to install more traffic measurement stations on road sections (increasing the possibility to estimate the traffic condition on road sections between the stations, based on the variability of traffic flow parameters). Another way to improve the quality of collected data is to use technologies that enable monitoring of the traffic flow condition and movement of individual vehicles along road sections. The most promising sensors that can be used in SSM-based research and current traffic safety management (e.g., for detecting incidents or dangerous driver behaviour) are RADAR sensors and video image processors (VIPs). The use of advanced algorithms in processing data from these sensors allows us to analyse the trajectory of individual vehicles on a given road section (vehicle position, dynamics of speed changes) and to detect interactions between vehicles. Such data were not available for the research presented in this paper. However, the impact of the information displayed on the VMS on driver behaviour required a detailed analysis of data on changes in driver behaviour (speed changes and the dynamics of these changes) along the road. Such analysis is currently possible only with the use of a driving simulator [97]. This approach made it possible to determine the areas where drivers decide to change speed as a result of seeing information on the VMS and further calibration of microscopic models. In future research works, we plan to take in-depth field measurements of vehicle trajectory and speed change dynamics in the vicinity of the VMS using RADAR sensors and video image processing techniques.

In the case of tests using a driving simulator, one measure of driver distraction may be lane-keeping information. The efficiency of lane-keeping by the driver (lateral control capability) can then be analysed. The literature reports point to two aspects of additional driver tasks, namely distraction, but also a possible increase in driver performance during a difficult task. The realisation of an additional task may be difficult in this case. The authors of the paper [69] concluded that the increase in cognitive load, which, although disruptive to the activities performed by the driver, causes an increase in lane-keeping performance. It seems that both cases described above can be observed as a result of experiments carried out under the RID-4D project. The histograms of lateral deviation values and histograms of standard deviation of this variable presented in this article may be used for preliminary evaluation of the analysed phenomena. The histograms present distributions of values close to the normal distribution, indicating the existence of value intervals with the number of values much not greater than the number of adjacent intervals. For the analysed cases, the mean value of the lane deviation and the variance of this variable were also calculated. In the analysed cases, a positive impact of ITS solutions on vehicle lane-keeping was noted. It varies from case to case, but in combination with other aspects of the application of ITS solutions, such actions aimed at improving road safety should be positively evaluated. The research also allowed us to define the drivers’ behaviours with the variable message signs, including the information provided by the signs. The results of the research conducted with the driving simulator were used to calibrate the microscopic models, examples of which are presented in this paper and are a promising source of data to develop models of driver behaviour.

This paper presents the methodology used for the preliminary assessment of the impact of a variable speed limit on major roads on the efficiency and safety of traffic on sections of expressways, taking into account the remaining elements of the test network model. The methodology is recommended to be used in planned implementations both to determine the effectiveness of planned solutions, their optimal location and the type of information provided to drivers, taking into account the traffic volume. The traffic safety assessment method is based on the analysis of surrogate traffic safety measures. In the presented case, TTC measures, representing the probability of accidents, and MaxDeltaV, determining their severity, were used. The above-mentioned measures are widely used in road safety analyses, however, to date the research has not been focused on such a broad approach as presented in this article, which takes into account not only the impact of measures on individual elements of the road network but also on the entire network of roads (major road corridor). In the presented studies, the Surrogate Safety Assessment Model (SSAM) was used for the first time to assess the safety of an ITS service, such as VSLs, with the use of surrogate safety measures. Added value is also found in the study and results of the location of variable message signs in the VSL system on a road section developed using the SSAM. Besides, an approach that takes into account the areas of conflict accumulation and conflict density in SSM modelling concerning traffic intensity seems promising.

Previous research on VSL systems [111,112], or co-operative VSL systems (C-VSLSs) [113,114,115] used simulation traffic models but were mostly conducted to assess traffic conditions without considering road safety. Yu and Abdel-Aty [116] found a level of safety that increased with VSL techniques on steep mountain bottlenecks using VISSIM microscopic simulation software. They estimated and used a real-time crush risk assessment model to quantify the crash risk. The safety impact of the VSL system was quantified as decreasing the risk of a crash and improving speed homogeneity. The conclusions showed that the proposed VSL system could improve road traffic safety. The results of our research also confirm the positive impact of VSLs on the level of traffic safety. The decrease in the number of conflicts and their severity indicates homogenisation of vehicle speed (TTC and MaxDeltaV measures reflect changes in speed differences between vehicles). This is particularly noticeable when using a series of VMSs on the road (39% reduction in the number of conflicts on major road sections, 52% reduction in the severity of conflicts) for rear-end conflicts compared to the baseline scenario without using VSLs. An even greater safety improvement was observed in the case of lane change conflicts (59% and 64%, respectively). The reduction in the number of lane change conflicts and their severity also shows the positive impact of VSLs on the homogenisation of traffic in terms of speed (less tendency for drivers to change lanes after speed harmonisation). Moreover, in our research, simulation models were calibrated using the driving simulator data and also real data on speed and time headway distribution from the traffic measurement stations. Therefore, the tendency of drivers to exceed the allowed speed on Polish roads was taken into account. This is important in terms of the effectiveness of VSL systems, as the results of research [116,117] indicated that the VSL system fails to significantly enhance traffic safety under low driver compliance.

Many of the issues mentioned in this paper indicate the possibility of developing in-depth research. In future research on VSLs, it would make sense to compare SSMs with the actual occurrence of incidents and to analyse real data (vehicle trajectories) obtained through RADAR sensors or image processing techniques. It will be important to take into account the provision of information to drivers by ways other than VMSs, e.g., in-vehicle information. Data from connected vehicles can be a new source of information for VSLs. Data on vehicle speed, distance to VMSs and position on the road can be used to improve VSL performance. If the number of connected vehicles is high enough, traffic management systems will not depend on detection stations and VMSs, which are expensive to maintain. Instead, traffic management systems could use V2V and V2I communication to provide drivers with information about individualised speed limits. The use of more data and the timely provision of information to the driver will improve traffic safety. It is important to make use of the fusion of data from many available sources (including sensors located in the vehicle and the road environment, as well as mobile devices or systems). It would also make sense to develop density analyses of different types of conflicts depending on the VSL solutions and to conduct studies on the impact of different speed control strategies and algorithms on road traffic safety on different types of roads. The research methodology presented in this paper and the developed research tools allow us to deepen the research in the areas mentioned above. The location of sensors collecting data for traffic control is also one of the key issues and should be thoroughly studied in future research work. The rational placement of sensors in the road network allows for continuous monitoring of traffic conditions and providing information to the driver at the right time. Providing information to the driver at an early stage reduces traffic disturbances and warns the driver about traffic hazards.

The problems presented in this article may provide advice on the development of sensor research to use the data for research on traffic safety management. Knowledge of what data are needed to manage and analyse traffic safety is essential to decide on the directions of sensor technology development. The presented studies have confirmed the positive impact of variable speed limits on the level of road safety and the efficiency of traffic. The impact may vary depending on the volume of traffic, the continuity of informing drivers about limits and the location of VMSs.

## Figures and Tables

**Table 1:** Cohorts defined for traffic intensity scenarios.

Cohort	Traffic Volumes in the Cohort q (veh/h /lane)	Volume-to-Capacity Ratio q/C	The Intensity of Traffic Assumed for the Load of the Major Road Lane in Test Models	Representative Volume-to-Capacity Ratio q/C
0	q > 2100	0.95–1	2150	0.98
1	1300–2099	0.59–0.95	1700	0.77
2	720–1299	0.33–0.59	1010	0.46
3	0–719	0–0.33	360	0.16

**Table 2:** Average value and the standard deviation of the distance between vehicle axis and lane axis.

No.	Scenario	Average Value of the Position Deviation (m)	Standard Deviation of Lateral Position (SDLP) (m)
1.	Static limit (S0/G5)	−0.063	0.307
2.	Speed limit on the Variable Message Sign (VMS) (S1/G2)	−0.134	0.332
3.	Speed limit on VMS with the reason for limitation (S2/G5)	−0.090	0.318

**Table 3:** Results of simulations for traffic safety assessment in the individual scenarios.

Part of Road Network	Type of Conflict	Measure	Scenario			Difference	
			W0	W1	W2	W1/W0	W2/W0
**Main road sections without merging and weaving sections**	crossing	Number of conflicts	0	0	0	0%	0%
	lane change	Number of conflicts	1183	886	489	−25%	−59%
		MaxDeltaV > 20 km/h	760	562	271	−26%	−64%
	rear end	Number of conflicts	4899	4228	2969	−12%	−39%
		MaxDeltaV > 20 km/h	210	184	100	−12%	−52%
**Interchanges along major roads**	crossing	Number of conflicts	226	254	205	12%	−9%
		MaxDeltaV > 20 km/h	223	252	200	13%	−10%
	lane change	Number of conflicts	699	675	657	−3%	−6%
		MaxDeltaV > 20 km/h	230	194	234	−16%	2%
	rear end	Number of conflicts	3604	3724	3500	3%	−3%
		MaxDeltaV > 20 km/h	192	188	191	−2%	−1%
**Other parts of the road network**	crossing	Number of conflicts	11	14	16	27%	45%
		MaxDeltaV > 20 km/h	1	2	9	100%	800%
	lane change	Number of conflicts	3	12	15	300%	400%
		MaxDeltaV > 20 km/h	0	1	0	100%	0%
	rear end	Number of conflicts	919	889	934	−3%	2%
		MaxDeltaV > 20 km/h	0	0	1	0%	100%
Number of conflicts			11,544	10,742	8785	−7%	−24%
MaxDeltaV > 20 km/h			1616	1383	1006	−14%	−38%

**Table 4:** Results of simulations for traffic efficiency assessment in individual scenarios.

Measure	W0	W1	W2	W1/W0	W2/W0
Average delays (s/veh)	85.35	82.98	73.46	−2.8%	−13.9%
Average number of stops (stops/veh)	0.42	0.43	0.40	3.0%	−4.8%
Mean speed (km/h)	64.52	64.30	63.60	−0.3%	−1.4%
Total delays in the entire network (h)	38,140	37,180	33,010	−2.5%	−13.5%
Total number of stops	65,888	68,313	63,279	3.7%	−4.0%

**Table 5:** Traffic efficiency measures for the entire test network for the model with an expressway (S 2/2).

Scenario		Traffic Volume (veh/h/lane)	Average Delays (s/veh)	Average Number of Stops	Average Speed (km/h)	Total Delay (h)	Total Number of Stops
W0	Without incident	1010	58.51	0.31	74.83	1563	28,988
W2			55.31	0.31	73.17	1498	28,984
W0		1700	85.35	0.42	64.52	3814	65,888
W2			73.46	0.40	63.60	3301	63,279
W0	With incident	1010	67.84	0.42	70.86	1904	40,899
W2			64.66	0.39	69.52	1838	38,512
W0		1700	91.90	0.68	62.07	4360	117,601
W2			85.89	0.67	60.04	4168	117,738

**Table 6:** Traffic safety measures for the entire test network for the test model with an expressway (S 2/2).

Scenario		Traffic Volume (veh/h/lane)	Number of Conflicts	MaxDeltaV >20 km/h	Number of Conflicts	MaxDeltaV >20 km/h
			Entire Test Network		Major Road	
W0	Without incident	1010	2079	446	984	303
W2			1940	374	816	233
W0		1700	11,544	1616	6082	970
W2			8785	1006	3458	371
W0	With incident	1010	3208	507	2012	361
W2			2978	428	1791	277
W0		1700	13,520	1511	8889	876
W2			13,007	1082	8252	447
